# HNCGAT: a method for predicting plant metabolite–protein interaction using heterogeneous neighbor contrastive graph attention network

**DOI:** 10.1093/bib/bbae397

**Published:** 2024-08-20

**Authors:** Xi Zhou, Jing Yang, Yin Luo, Xiao Shen

**Affiliations:** School of Tropical Agriculture and Forestry, Hainan University, 58 Renmin Avenue, Haikou 570228, Hainan, China; School of Tropical Agriculture and Forestry, Hainan University, 58 Renmin Avenue, Haikou 570228, Hainan, China; School of Tropical Agriculture and Forestry, Hainan University, 58 Renmin Avenue, Haikou 570228, Hainan, China; School of Computer Science and Technology, Hainan University, 58 Renmin Avenue, Haikou 570228, Hainan, China

**Keywords:** metabolite–protein interactions, heterogeneous graph neural network, neighbor contrastive learning

## Abstract

The prediction of metabolite–protein interactions (MPIs) plays an important role in plant basic life functions. Compared with the traditional experimental methods and the high-throughput genomics methods using statistical correlation, applying heterogeneous graph neural networks to the prediction of MPIs in plants can reduce the cost of manpower, resources, and time. However, to the best of our knowledge, applying heterogeneous graph neural networks to the prediction of MPIs in plants still remains under-explored. In this work, we propose a novel model named heterogeneous neighbor contrastive graph attention network (HNCGAT), for the prediction of MPIs in *Arabidopsis*. The HNCGAT employs the type-specific attention-based neighborhood aggregation mechanism to learn node embeddings of proteins, metabolites, and functional-annotations, and designs a novel heterogeneous neighbor contrastive learning framework to preserve heterogeneous network topological structures. Extensive experimental results and ablation study demonstrate the effectiveness of the HNCGAT model for MPI prediction. In addition, a case study on our MPI prediction results supports that the HNCGAT model can effectively predict the potential MPIs in plant.

## Introduction

The metabolites are the intermediate or end products of the metabolism process, which are usually produced by humans, plants, and microbes [1]. In plants, there are >200 000 metabolites of different structures [2]. Plant metabolites are directly involved in various basic life functions, such as respiration, photosynthesis, cell division, growth, storage, and reproduction, through interacting with specific proteins [3]. With the help of metabolite–protein interactions (MPIs), plants can adapt to their environments and thrive in their surroundings [4]. Moreover, plant metabolites can serve as important materials for food and cosmetic industrial products [5, 6], and have been widely used in medical/pharmacological applications [7].

Our understanding of MPIs in plants is very limited compared with the interactions between proteins or interactions between proteins and DNA [8]. This is attributed to the complexity of MPIs and the difficulty in identifying the players in the interactions [9]. Classical biochemistry and genetics still do not allow large-scale identification of MPIs [10]. In recent years, more and more biochemical methods have been developed for identifying MPIs, such as tandem affinity purification [11], affinity chromatography [11], ligand-detecting nuclear magnetic resonance [12], yeast three-hybrid [12], limited proteolysis-coupled mass spectrometry [13], protein–metabolite interactions with size separation [14], etc. These biochemical methods are relatively accurate, but costly in terms of materials and time consumption [15]. Recently, new high-throughput metabolome–transcriptome co-analysis methods have been developed, which utilize the statistical correlation of the expression of metabolites in the metabolome and genes in the transcriptome, in different conditions or tissues, to establish associations between them [16, 17]. However, the aforementioned high-throughput methods of joint metabolome and transcriptome analysis can only establish the correlation between metabolites and proteins, which does not indicate that they have interactions. In addition, the accuracy of such high-throughput methods has not been systematically evaluated, and they also require a lot of human and material resources.

Heterogeneous graphs (also known as heterogeneous information networks) [18], containing different types of entities (i.e. nodes) and/or different types of relations (i.e. edges), have become ubiquitous in real-world scenarios, ranging from social networks, electronic health records networks, to biological networks. Heterogeneous graph neural networks are designed to learn low-dimensional embeddings for heterogeneous graphs, which can preserve complex heterogeneous topological structures and semantics for various downstream tasks, including node classification, node clustering, and link prediction. Heterogeneous graph neural networks have been successfully applied to a wide range of tasks in biological and medical domains, such as the prediction of drug–drug interactions, drug–target interactions, drug–disease associations, lncRNA–disease associations, and so on. For example, Luo *et al*. [19] introduced DTINet, which integrates diverse drug-related information to predict novel drug–target interactions from a constructed heterogeneous network. Wan *et al*. [20] proposed a NeoDTI framework that leverages diverse information from heterogeneous network data to learn representations of drugs and targets for the prediction of drug–target interactions. Gao *et al*. [21] proposed a similarity measures-based graph co-contrastive learning model to learn embeddings from heterogeneous network topologies for the prediction of drug–disease associations. Shi *et al*. [22] constructed a heterogeneous network composed of lncRNA similarity network, lncRNA–disease association network, and lncRNA–miRNA association network, and proposed a heterogeneous graph neural network with type-specific neighborhood aggregation functions to learn embeddings to predict potential lncRNA–disease associations. Rafiei *et al*. [23] proposed CFSSynergy, which utilized transformer-based architecture for drugs and created a similarity matrix between proteins using the Node2Vec algorithm, for drug synergy prediction. Dehghan *et al*. [24] used multimodal knowledge as input and proposed an attention-based fusion technique to combine this knowledge for drug–target interaction prediction. Zhao *et al*. [25] proposed a deep-learning model that focused on the representation of sequence encoding and variational information bottlenecks for peptide toxicity prediction. Zhang *et al*. [26] constructed EmerGNN, a graph neural network that can effectively predict interactions for emerging drugs by leveraging the rich information in biomedical networks.

Inspired by the recent success of heterogeneous graph neural networks on the prediction of drug–target interactions and drug–disease associations, our work aims to apply heterogeneous graph neural networks to predict potential MPIs in plants. Compared with the traditional high-precision and low-throughput biochemical experimental methods and the high-throughput genomics methods using statistical correlation, applying heterogeneous graph neural networks to the prediction of MPIs in plants can not only fill in the lack of data in the existing databases, but also dramatically reduce the cost of manpower, resources, and time, which is of great research value. However, to the best of our knowledge, applying heterogeneous graph neural networks to the prediction of MPIs in plants still remains under-explored up to now. This is mainly attributed to two challenges. First, it is unclear how to construct a heterogeneous network for MPIs in plants, i.e. what types of nodes and edges should be included. Unlike the abundant research on the prediction of drug–target interactions and drug–disease associations, the MPIs prediction in plants suffers from the lack of public benchmark dataset. Thus, the first challenge (CH1) is how to collect and pre-process the multimodal data to construct a heterogeneous networked dataset for MPIs. Then, upon the constructed heterogeneous network with multiple types of nodes and edges, the second challenge (CH2) is how to develop a heterogeneous graph neural network to integrate the complex multi-relational topological structures and multimodal semantics to learn informative embeddings for effective prediction of potential MPIs.

In this work, we focus on the MPI prediction in *Arabidopsis* [27], a model organism of plants. To address CH1, we construct an *Arabidopsis* heterogeneous network by collecting the multimodal information from multiple publicly available databases. The *Arabidopsis* heterogeneous network composed of three types of nodes, i.e. proteins, metabolites, and functional-annotations, and five types of edges including metabolite–protein-interactions, metabolite–functional-annotation-associations, metabolite–metabolite-structural-similarities, protein–protein-interactions, and protein–functional-annotation-associations.

On the other hand, to address CH2, we propose a novel model, named heterogeneous neighbor contrastive graph attention network (HNCGAT) for MPI prediction in *Arabidopsis*. First, we employ a heterogeneous graph attention network to learn node embeddings by adaptively aggregating neighborhood embeddings w.r.t. each specific edge type. Then, different edge-type-specific node embeddings are concatenated as the final node embeddings for each type of nodes, i.e. proteins, metabolites, and functional-annotations. Second, we design a novel heterogeneous neighbor contrastive learning framework to guide the node embedding learning to maximize the mutual information between positive pairs and minimize that between negative pairs across heterogeneous networks. Here, the heterogeneous network topological structures are taken as the supervised signals to define positive and negative pairs. Specifically, for each anchor node, it forms positive pairs with various types of its neighbors and negative pairs with various types of its non-neighbors across heterogeneous networks. As a result, the heterogeneous neighborhood information can be well preserved by the learned node embeddings, which is conductive for link prediction between metabolites and proteins.

The contributions of this work are summarized as follows:

1) To the best of our knowledge, we are the first to study the problem of MPI prediction in *Arabidopsis* by taking advantage of heterogeneous graph neural networks. To tackle this problem, we construct the first *Arabidopsis* heterogeneous networked dataset, which integrates multimodal information from three types of nodes and five types of edges.
2) We propose a novel HNCGAT model for MPI prediction in *Arabidopsis*. The proposed HNCGAT employs the type-specific attention-based neighborhood aggregation mechanism to learn node embeddings. Moreover, HNCGAT designs a novel neighbor contrastive learning framework to guide node embedding learning to preserve the heterogeneous network topologies for effective MPI prediction.
3) Extensive experimental results and ablation study demonstrate the effectiveness of the proposed HNCGAT for MPI prediction in *Arabidopsis*. In addition, a case study on our MPI prediction results supports that the proposed HNCGAT model can effectively predict the potential MPIs, which have not yet included in the public plant metabolite database AraCyc, but have been identified by the recent methods in the literature.

## Materials and methods

### Dataset

**Figure 1:**
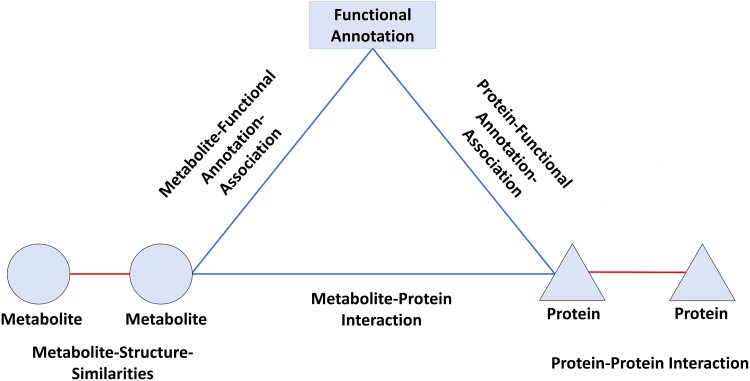
Overview of the *Arabidopsis* heterogeneous network in our study.

In this work, we formulate the problem of MPI prediction as a metabolite–protein link prediction task using multimodal interaction data. We construct an Arabidopsis heterogeneous network using multiple public databases. Figure 1 shows the overview of the Arabidopsis heterogeneous network, which contains three types of nodes and five types of edges, representing the multimodal information related to metabolite–protein associations. Table 1 illustrates the statistics and the sources of *Arabidopsis* heterogeneous networked dataset.

**Table 1:** The statistics of *Arabidopsis* heterogeneous networked dataset

	Type	Count	Sources
Nodes	Metabolite (M)	1331	AraCyc
	Protein (P)	12 641	TAIR
	Functional-annotation (F)	7681	GO
Edges	Metabolite–protein-interaction (MP)	20 515	AraCyc
	Metabolite–metabolite-structure-similarity (MM)	613 277	Rdkit
	Metabolite–functional-annotation-association (MF)	6740	AraCyc, GO
	Protein–protein-interaction (PP)	49 614	BioGrid
	Protein–functional-annotation-association (PF)	80 398	GO, TAIR

#### Three types of nodes

The Arabidopsis heterogeneous network contains three types of nodes. (i) The metabolite nodes were extracted from the AraCyc database (version 17.0) [28]. AraCyc is a regularly updated database containing Arabidopsis pathways and their catalytic enzymes, compounds, and genes. The majority of pathway diagrams in AraCyc were manually extracted from the plant literature. They are either supported by experimental evidence or based on expert hypotheses [29]. (ii) The protein nodes were extracted from the biggest Arabidopsis resource database, The Arabidopsis Information Resource (TAIR) [30], which maintains a database of genetic and molecular biology data for the model higher plant *Arabidopsis thaliana*. (iii) The functional-annotation nodes were representing Gene Ontology terms (GO terms) and extracted from the GO database [31]. The GO database is the world’s largest source of information on the functions of genes.

#### Five types of edges

The *Arabidopsis* heterogeneous network contains five types of edges. (i) Metabolite–protein-interaction: In AraCyc database (version 17.0) [28], each metabolite was assigned to a pathway, which has a Reaction ID or EC ID, and each experimentally verified enzyme and gene (i.e. protein) was also assigned to pathways. Thus, using the same Reaction ID or the same EC ID as an indicator, we can extract 20 515 MPIs between 1216 metabolites and 3459 proteins. Most of metabolites (60%) interact with 5 proteins; only 10% of metabolites interact with >30 proteins. A total of 90% of proteins have interaction with <5 metabolites; 1% of proteins interact with >20 metabolites. (ii) Metabolite–functional-annotation-association: In GO database [31], Reaction ID or EC ID was annotated to a GO term. With this annotation, we extracted 6740 metabolite–functional-annotation-associations that covers 1158 metabolites and 1346 functional-annotations. (iii) Metabolite–metabolite-structure-similarity: To measure the pair-wise metabolite structure similarity, we adopted the dice similarities of the Morgan fingerprints with radius 2 [32], with the SMILES format using the RDKit. The SMILES format of each metabolite is extracted from AraCyc database. Due to the lack of SMILES format, we finally extracted 1 226 554 metabolite–metabolite-structure-similarities between 1108 metabolites. (iv) Protein–protein-interaction: The *Arabidopsis* protein–protein-interactions were downloaded and extracted from the BioGrid (version 4.2.191) [33], resulting in 49 614 protein–protein-interactions between 10 478 proteins. (v) Protein–functional-annotation-association: The protein–functional-annotation-associations were imported from GO database [34]. GO annotations are created by associating a gene or gene product with a GO term. We excluded the annotations that is with the GO Evidence Code of IEA, and obtained 80 398 protein–functional-annotation-associations between 12 549 proteins and 6205 functional-annotation.

#### Problem definition

An *Arabidopsis* heterogeneous network can be represented by an undirected multi-relational graph $\mathcal{G}=\left(\mathcal{V},\mathcal{E}\right)$. The graph $\mathcal{G}$ contains three types of nodes, i.e. ${\mathcal{V}}^P$ is a set of protein nodes, ${\mathcal{V}}^M$ is a set of metabolite nodes, and ${\mathcal{V}}^F$ is a set of functional-annotation nodes. In addition, the graph $\mathcal{G}$ consists of five types of edges, including metabolite-protein-interactions ${\mathcal{E}}^{MP}$, metabolite–functional-annotation-associations ${\mathcal{E}}^{MF}$, protein–functional-annotation-associations ${\mathcal{E}}^{PF}$, metabolite–metabolite-structure-similarities ${\mathcal{E}}^{MM}$, and protein–protein-interactions ${\mathcal{E}}^{PP}$. For MPI prediction on the *Arabidopsis* heterogeneous network, a fraction of edges between metabolites and proteins in ${\mathcal{E}}^{MP}$ are sampled to construct the training set ${\mathcal{E}}_{tr}^{MP}$, while the remaining edges in ${\mathcal{E}}^{MP}$ are employed to construct the test set ${\mathcal{E}}_{te}^{MP}$. During model training, only the edges in the training set ${\mathcal{E}}_{tr}^{MP}$ are observed, and the goal of MPI prediction is to predict the edges in the test set ${\mathcal{E}}_{te}^{MP}$, by taking advantage of the multimodal information of the *Arabidopsis* heterogeneous network. The frequently used notations are summarized in Table 2.

**Table 2:** Frequently used notations

Notations	Descriptions
$\mathcal{G}$	An *Arabidopsis* heterogeneous network
${v}_i^P$ *,* ${v}_i^M,{v}_i^F$	*i*th node of protein, metabolite, and functional annotation
$\left({v}_i^M,{v}_j^P\right)$	An edge connecting a metabolite node ${v}_i^M$ and a protein node ${v}_j^P$
${h}_i^P$ , ${h}_i^M$, ${h}_i^F$	Final embedding vector of protein node ${v}_i^P$, metabolite node ${v}_i^M$*,* and functional annotation node ${v}_i^F$
${e}_i^{PM}$ , ${e}_i^{PP}$, ${e}_i^{PF}$	The aggregated neighborhood embedding of protein node ${v}_i^P$ from its metabolite, protein, and functional annotation neighbors
${e}_i^{MM}$ , ${e}_i^{MP}$, ${e}_i^{MF}$	The aggregated neighborhood embedding of metabolite node ${v}_i^M$ from its metabolite, protein, and functional annotation neighbors
${e}_i^{FP}$ , ${e}_i^{FM}$	The aggregated neighborhood embedding of functional annotation node ${v}_i^F$ from its protein and metabolite neighbors
${x}_i^P$ , ${x}_i^M$, ${x}_i^F$	The initial randomized attribute vector of protein node ${v}_i^P$, metabolite node ${v}_i^M$*,* and functional annotation node ${v}_i^F$
${\mathcal{N}}_i^{MP}$ , ${\mathcal{N}}_i^{PP}$, ${\mathcal{N}}_i^{FP}$	The set of observed metabolite, protein, and functional annotation neighbors of protein ${v}_i^P$
${\mathcal{N}}_i^{MM}$ , ${\mathcal{N}}_i^{PM}$, ${\mathcal{N}}_i^{FM}$	The set of observed metabolite, protein, and functional annotation neighbors of metabolite ${v}_i^M$
${\mathcal{N}}_i^{MF}$ , ${\mathcal{N}}_i^{PF}$	The set of observed metabolite, protein neighbors of functional annotation ${v}_i^F$
$\ell \left({h}_i^P\right)$ , $\ell \left({h}_i^M\right)$, $\ell \left({h}_i^F\right)$	The heterogeneous neighbor contrastive loss associated with the anchor protein, metabolite, and functional-annotation embedding ${h}_i^P$, ${h}_i^M$, ${h}_i^F$, respectively
${h}_{\left({v}_i^M,{v}_j^P\right)}$	The edge embedding vector of $\left({v}_i^M,{v}_j^P\right)$

#### Heterogeneous neighbor contrastive graph attention network

**Figure 2:**
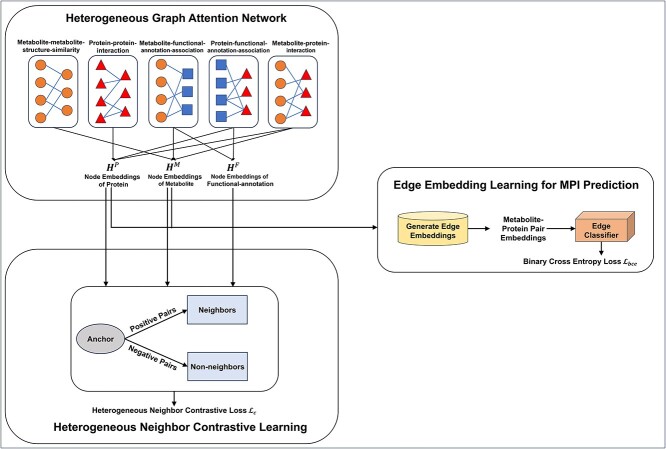
The model architecture of HNCGAT, which contains (i) heterogeneous graph attention network for node embedding learning, (ii) heterogeneous neighbor contrastive learning to preserve heterogeneous network topology, and (iii) edge embedding learning for MPI prediction.

Next, we develop a novel framework, HNCGAT, to automatically learn various types of node embeddings from the *Arabidopsis* heterogeneous network $\mathcal{G}$ that can be used to facilitate the prediction of MPIs. Figure 2 shows the model architecture of HNCGAT. It first employs a heterogeneous graph attention network to learn node embeddings of proteins, metabolites, and functional-annotations, by the type-specific attention-based neighborhood aggregation mechanism. Then, it applies heterogeneous neighbor contrastive learning to map each node close to its heterogeneous neighbors, while far away from its heterogeneous non-neighbors. As a result, the heterogeneous network topologies can be well preserved by the learned node embeddings. Finally, an edge classifier is adopted for MPI prediction, by taking the learned node embeddings of proteins and metabolites as the input.

#### Heterogeneous graph attention network for node embedding learning

Node embeddings of proteins are learned by integrating the multimodal information in the protein–protein-interaction network, metabolite–protein-interaction network, and protein–functional-annotation-association network. Specifically, to learn the embedding of a protein node ${v}_i^P$, we concatenate four types of embeddings, (i) the embedding ${e}_i^P$ learned from its initial protein attributes, (ii) the embedding ${e}_i^{PP}$ learned from its associated topological structure in the protein–protein-interaction network, (iii) the embedding ${e}_i^{PM}$ aggregated from its observed metabolite neighbors in the training protein–metabolite network, and (iv) the embedding ${e}_i^{PF}$ aggregated from its functional-annotation neighbors in the protein–functional-annotation network, which are expressed as follows: 


(1) \begin{equation*} {h}_i^P=\left[{e}_i^{\mathrm{P}}\left\Vert{e}_i^{PP}\right.\left\Vert{e}_i^{PM}\right.\left\Vert{e}_i^{PF}\right.\right] \end{equation*}

where ${h}_i^P$ denotes the final embedding vector of a protein node ${v}_i^P$, and $\left[\bullet \left\Vert \bullet \right.\right]$denotes the concatenation operation.

First, the embeddings ${e}_i^P$ and ${e}_i^{PP}$ in Equation (1) are learned by the single-layer perceptron, as follows:


(2) \begin{equation*} {e}_i^P=\mathrm{ReLU}\left({W}^P{x}_i^P\right) \end{equation*}

(3) \begin{equation*} {e}_i^{PP}=\mathrm{ReLU}\left({W}^{PP}{a}_i^{PP}\right) \end{equation*}

where ${x}_i^P$ is the initial randomized protein attribute vector of ${v}_i^P$, ${a}_i^{PP}={A}^{PP}\left(i,:\right)$ is the input topological structure vector of ${v}_i^P$ in the protein–protein-interaction network, which is the $i$th row of the protein–protein-interaction matrix ${A}^{PP}$, specifically, ${A}_{ij}^{PP}$=1 if $\left({v}_i^P,{v}_j^P\right)\in{\mathcal{E}}^{PP}$, i.e. protein ${v}_i^P$ interacts with protein ${v}_j^P$; otherwise ${A}_{ij}^{PP}$=0. ${W}^P$ and ${W}^{PP}$ are the learnable weight matrices.

The embedding ${e}_i^{PM}$ in Equation (1) is learned by a bipartite graph attention layer, which adaptively aggregates the attributes of the observed metabolite neighbors of ${v}_i^P$ and then concatenates the embedding vector of protein attributes of ${v}_i^P$, which is expressed as follows: 


(4) \begin{equation*} {e}_i^{PM}=\mathrm{ReLU}\left(\left[{e}_i^P\left\Vert{\sum}_{j\in{\mathcal{N}}_i^{MP}}{\alpha}_{i,j}^{PM}{W}^M{x}_j^M\right.\right]\right) \end{equation*}

where ${\mathcal{N}}_i^{MP}=\left\{{v}_j^M\in{\mathcal{V}}^M|\left({v}_i^P,{v}_j^M\right)\in{\mathcal{E}}_{tr}^{MP}\right\}$ is a set of observed metabolite neighbors of protein ${v}_i^P$ connected by the training edges ${\mathcal{E}}_{tr}^{MP}$ in the metabolite–protein network, ${\boldsymbol{x}}_j^M$denotes the initial randomized metabolite attribute vector of ${v}_j^M$, ${W}^M$ is the learnable weight matrix. In addition, ${\alpha}_{i,j}^{PM}$ denotes the attention weight of edge $\left({v}_i^P,{v}_j^M\right)$ between protein ${v}_i^P$ and metabolite ${v}_j^M$, normalized among ${\mathcal{N}}_i^{MP}$, which is learned as:


(5) \begin{equation*} {\alpha}_{i,j}^{PM}=\frac{\exp \left(\mathrm{LeakyReLU}\left(\boldsymbol{\varphi} \left[{\boldsymbol{W}}^P{\boldsymbol{x}}_i^P\left\Vert{\boldsymbol{W}}^M{\boldsymbol{x}}_j^M\right.\right]\right)\right)}{\sum_{k\in{\mathcal{N}}_i^{MP}}\exp \left(\mathrm{LeakyReLU}\left(\boldsymbol{\varphi} \left[{\boldsymbol{W}}^P{\boldsymbol{x}}_i^P\left\Vert{\boldsymbol{W}}^M{\boldsymbol{x}}_k^M\right.\right]\right)\right)} \end{equation*}

where $\varphi$ is the learnable weight vector. The embedding ${e}_i^{PF}$ is learned by a bipartite graph attention layer similar to Equation (4), which adaptively aggregates the attributes of the functional-annotation neighbors of ${v}_i^P$ and then concatenates the embedding vector of protein attributes of ${v}_i^P$.

Node embeddings of metabolites are generated by utilizing the multimodal information in metabolite–protein-interaction network, metabolite–functional-annotation-association network, and metabolite–metabolite-structure-similarity network. Specifically, the embedding of a metabolite node ${v}_i^M$ is learned as:


(6) \begin{equation*} {h}_i^M=\left[{e}_i^M\left\Vert{e}_i^{MM}\right.\left\Vert{e}_i^{MP}\right.\left\Vert{e}_i^{MF}\right.\right] \end{equation*}

where the embedding ${e}_i^M$ is learned from the initial randomized metabolite attributes, and the embedding ${e}_i^{MM}$ is learned from its associated structural similarity with other metabolites by the single-layer perceptron similar to Equations (2) and (3), respectively. In addition, the embeddings ${e}_i^{MP}$ and ${e}_i^{MF}$ are aggregated from its observed protein neighbors and its functional-annotation neighbors by the bipartite graph attention layer similarly as in Equation (4).

Node embeddings of functional-annotations are learned by leveraging the multimodal information in the metabolite–functional-annotation-association network and protein–functional-annotation-association network. Specifically, the embedding of a functional-annotation node ${v}_i^F$ is formulated as follows:


(7) \begin{equation*} {h}_i^F=\left[{e}_i^F\left\Vert{e}_i^{FP}\right.\left\Vert{e}_i^{FM}\right.\right] \end{equation*}

where the embedding ${e}_i^F$ is learned from the initial randomized functional-annotation attributes by a single-layer perceptron similar to Equation (2), the embeddings ${e}_i^{FP}$ and ${e}_i^{FM}$ are aggregated from its protein neighbors and metabolite neighbors, respectively, by the bipartite graph attention layer similar to Equation (4).

#### Heterogeneous neighbor contrastive learning for persevering heterogeneous network topology

Graph contrastive learning [35], as an effective self-supervised graph representation learning technique, has demonstrated outstanding performance on learning informative node embeddings to facilitate various downstream tasks, such as node classification and link prediction. The latest study in homogeneous graph contrastive learning [36] proposes to utilize the network topological structure as the supervised signals to define positive pairs and negative pairs, where positive pairs are formed between the anchor and its neighbors within the same view and from the other view, negative pairs are formed between the anchor and its non-neighbors within the same view and from the other view. By pulling positive pairs together while pushing negative pairs far apart, the network topology can be well preserved by the learned node embeddings to facilitate the downstream tasks.

Inspired by [36], our work customizes a heterogeneous neighbor contrastive learning framework to guide the node embedding learning of proteins, metabolites, and functional-annotations in the *Arabidopsis* heterogeneous network. Specifically, we consider the metabolite–protein-interaction network, metabolite–functional-annotation-association network, protein–functional-annotation-association network, metabolite–metabolite-structure-similarity network, and protein–protein-interaction network as different contrastive views. Then, each anchor node forms positive pairs with its heterogeneous neighbors, while forms negative pairs with its heterogeneous non-neighbors across different contrastive views. By optimizing the heterogeneous graph contrastive loss, the learned node embeddings of proteins, metabolites, and functional-annotations can effectively preserve the heterogeneous network topologies to facilitate the downstream MPI prediction.

**Figure 3:**
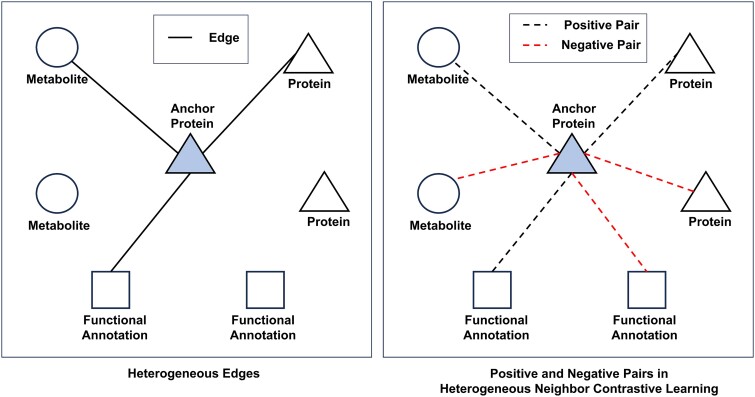
An illustration of positive and negative pairs defined in heterogeneous neighbor contrastive learning, given a protein node as the anchor.

First, given the embedding of a protein ${v}_i^P$ as the anchor, it forms positive pairs with three types of neighbors across different contrastive views, i.e. (i) its observed metabolite neighbors ${\mathcal{N}}_i^{MP}$ in the training protein–metabolite network, (ii) its protein neighbors ${\mathcal{N}}_i^{PP}$ in the protein–protein-interaction networ k, and (iii) its functional-annotation neighbors ${\mathcal{N}}_i^{FP}$in the protein–functional-annotation network. In contrast, the anchor protein ${v}_i^P$ forms negative pairs with two types of non-neighbors across different contrastive views, i.e. (i) its non-neighbors of proteins that are disconnected with ${v}_i^P$ in the protein–protein-interaction network, and (ii) its non-neighbors of functional-annotations that are disconnected with ${v}_i^P$ in the protein–functional-annotation network. Figure 3 illustrates the definition of positive and negative pairs by selecting a protein node as the anchor. The anchor protein forms positive pairs with its heterogeneous neighbors of protein, metabolite, and functional-annotation, while forms negative pairs with its heterogeneous non-neighbors.

According to the aforementioned positive and negative pairs, the heterogeneous neighbor contrastive loss associated with the anchor protein embedding ${h}_i^P$ is defined, by extending the normalized temperature-scaled cross-entropy loss (NT-Xent) loss [37] as:


$$ \ell \left({h}_i^P\right)=\qquad\qquad\qquad\qquad\qquad\qquad\qquad\qquad\qquad\qquad\qquad\qquad\qquad $$

\begin{equation*} -\log \frac{\left(\sum_{j\in{\mathcal{N}}_i^{MP}}\mathrm{sim}\left({h}_i^P,{h}_j^M\right)+\sum_{j\in{\mathcal{N}}_i^{PP}}\mathrm{sim}\left({h}_i^P,{h}_j^P\right)+\sum_{j\in{\mathcal{N}}_i^{FP}}\mathrm{sim}\left({h}_i^P,{h}_j^F\right)\right)/\left(\left|{\mathcal{N}}_i^{MP}\right|+\left|{\mathcal{N}}_i^{PP}\right|+\left|{\mathcal{N}}_i^{FP}\right|\right)}{\left(\underset{\mathrm{Positive}\ \mathrm{pairs}}{\underbrace{\sum_{j\in{\mathcal{N}}_i^{MP}}\mathrm{sim}\left({h}_i^P,{h}_j^M\right)+\sum_{j\in{\mathcal{N}}_i^{PP}}\mathrm{sim}\left({h}_i^P,{h}_j^P\right)+\sum_{j\in{\mathcal{N}}_i^{FP}}\mathrm{sim}\left({h}_i^P,{h}_j^F\right)}}\right)+\left(\underset{\mathrm{Negative}\ \mathrm{pairs}}{\underbrace{\sum_{j\notin{\mathcal{N}}_i^{PP}}\mathrm{sim}\left({h}_i^P,{h}_j^P\right)+\sum_{j\notin{\mathcal{N}}_i^{FP}}\mathrm{sim}\left({h}_i^P,{h}_j^F\right)}}\right)}\qquad\qquad\qquad\qquad\qquad\\ \quad\qquad(8) \end{equation*}

where $\mathrm{sim}\left(u,v\right)=\exp \left(\frac{u^Tv}{{\left\Vert u\right\Vert}_2{\left\Vert v\right\Vert}_2}/\tau \right)$ and $\tau$ is a temperature parameter. Minimizing Equation (8) would maximize the agreement between positive pairs by pulling them close and minimize the agreement between negative pairs by pushing them far apart.

It is worth noting that in Equation (8), we do not consider the unobserved metabolite neighbors, which are disconnected with the anchor protein by the training protein–metabolite edges in ${\mathcal{E}}_{tr}^{PM}$, as the negative samples. Since such unobserved metabolite neighbors might be connected with the anchor protein by the testing protein–metabolite edges in ${\mathcal{E}}_{te}^{PM}$, in such as case, considering them as negative samples would undesirably push them far away from the anchor protein, negatively affecting the prediction of MPIs on the *Arabidopsis* heterogeneous network.

Similarly, by selecting the embedding of a metabolite ${v}_i^M$ as the anchor, it forms positive pairs with three types of neighbors across different contrastive views, i.e. (i) its observed protein neighbors ${\mathcal{N}}_i^{PM}$ in the training protein–metabolite network, (ii) its metabolite neighbors ${\mathcal{N}}_i^{MM}$ in the metabolite–metabolite-structure-similarity network, and (iii) its functional-annotation neighbors ${\mathcal{N}}_i^{FM}$in the metabolite–functional-annotation network. And the anchor metabolite ${v}_i^M$ forms negative pairs with two types of non-neighbors across different contrastive views, i.e. (i) its non-neighbors of metabolites in the metabolite–metabolite-structure-similarity, and (ii) its non-neighbors of functional-annotations in the metabolite–functional-annotation network. Accordingly, the heterogeneous neighbor contrastive loss associated with the anchor metabolite embedding ${h}_i^M$ is defined as: 


$$ \ell \left({h}_i^M\right)=\qquad\qquad\qquad\qquad\qquad\qquad\qquad\qquad\qquad\qquad\qquad\qquad\qquad $$

(9) \begin{equation*} -\log \frac{\left(\sum_{j\in{\mathcal{N}}_i^{PM}}\mathrm{sim}\left({h}_i^M,{h}_j^P\right)+\sum_{j\in{\mathcal{N}}_i^{MM}}\mathrm{sim}\left({h}_i^M,{h}_j^M\right)+\sum_{j\in{\mathcal{N}}_i^{FM}}\mathrm{sim}\left({h}_i^M,{h}_j^F\right)\right)/\left(\left|{\mathcal{N}}_i^{MM}\right|+\left|{\mathcal{N}}_i^{PM}\right|+\left|{\mathcal{N}}_i^{FM}\right|\right)}{\left(\underset{\mathrm{Positive}\ \mathrm{pairs}}{\underbrace{\sum_{j\in{\mathcal{N}}_i^{PM}}\mathrm{sim}\left({h}_i^M,{h}_j^P\right)+\sum_{j\in{\mathcal{N}}_i^{MM}}\mathrm{sim}\left({h}_i^M,{h}_j^M\right)+\sum_{j\in{\mathcal{N}}_i^{FM}}\mathrm{sim}\left({h}_i^M,{h}_j^F\right)}}\right)+\left(\underset{\mathrm{Negative}\ \mathrm{pairs}}{\underbrace{\sum_{j\notin{\mathcal{N}}_i^{MM}}\mathrm{sim}\left({h}_i^M,{h}_j^M\right)+\sum_{j\notin{\mathcal{N}}_i^{FM}}\mathrm{sim}\left({h}_i^M,{h}_j^F\right)}}\right)}\qquad\qquad\qquad\qquad\qquad\quad(9) \end{equation*}

Finally, given the embedding of a functional-annotation ${v}_i^F$ as the anchor, it forms positive pairs with two types of neighbors across different contrastive views, i.e. (i) its protein neighbors ${\mathcal{N}}_i^{PF}$ in the protein–functional-annotations network, (ii) its metabolite neighbors ${\mathcal{N}}_i^{MF}$ in the metabolite–functional-annotation network. And the anchor functional-annotation ${v}_i^F$ forms negative pairs with two types of non-neighbors across different contrastive views, i.e. (i) its non-neighbors of proteins in the protein–functional-annotations network, and (ii) its non-neighbors of metabolites in the metabolite–functional-annotation network. Accordingly, the heterogeneous neighbor contrastive loss associated with the anchor functional-annotation embedding ${h}_i^F$ is defined as: 


$$ \ell \left({h}_i^F\right)=\qquad\qquad\qquad\qquad\qquad\qquad\qquad\qquad\qquad\qquad\qquad\qquad\qquad $$

(10) \begin{equation*} -\log \frac{\left(\sum_{j\in{\mathcal{N}}_i^{MF}}\mathrm{sim}\left({h}_i^F,{h}_j^M\right)+\sum_{j\in{\mathcal{N}}_i^{PF}}\mathrm{sim}\left({h}_i^F,{h}_j^P\right)\right)/\left(\left|{\mathcal{N}}_i^{MF}\right|+\left|{\mathcal{N}}_i^{PF}\right|\right)}{\left(\underset{\mathrm{Positive}\ \mathrm{pairs}}{\underbrace{\sum_{j\in{\mathcal{N}}_i^{MF}}\mathrm{sim}\left({h}_i^F,{h}_j^M\right)+\sum_{j\in{\mathcal{N}}_i^{PF}}\mathrm{sim}\left({h}_i^F,{h}_j^P\right)}}\right)+\left(\underset{\mathrm{Negative}\ \mathrm{pairs}}{\underbrace{\sum_{j\notin{\mathcal{N}}_i^{MF}}\mathrm{sim}\left({h}_i^F,{h}_j^M\right)+\sum_{j\notin{\mathcal{N}}_i^{PF}}\mathrm{sim}\left({h}_i^F,{h}_j^P\right)}}\right)}\qquad\qquad\qquad\qquad\qquad\qquad\qquad\qquad\qquad\qquad\ \ \quad(10) \end{equation*}

The total heterogeneous neighbor contrastive loss ${\mathcal{L}}_c$, averaged over all anchor nodes in proteins, metabolites, and functional-annotations is defined as follows:


(11) \begin{equation*} {\mathcal{L}}_c=\left(\frac{1}{\left|{\mathcal{V}}^P\right|}\sum_{v_i^P\in{\mathcal{V}}^P}\ell \left({h}_i^P\right)+\frac{1}{\left|{\mathcal{V}}^M\right|}\sum_{v_i^M\in{\mathcal{V}}^M}\ell \left({h}_i^M\right)+\frac{1}{\left|{\mathcal{V}}^F\right|}\sum_{v_i^F\in{\mathcal{V}}^F}\ell \left({h}_i^F\right)\right)/3 \end{equation*}

where $\left|{\mathcal{V}}^P\right|$, $\left|{\mathcal{V}}^M\right|$, and $\left|{\mathcal{V}}^F\right|$ are the number of nodes of protein, metabolite, and functional-annotation, respectively. Minimizing ${\mathcal{L}}_c$, each anchor node would be mapped close to its heterogeneous neighbors while far away from its heterogeneous non-neighbors across different contrastive views in the *Arabidopsis* heterogeneous network. As a result, the learned embeddings of proteins, metabolites, and functional-annotations can well preserve the heterogeneous network topological structures of different edge types, which is conductive for the downstream MPI prediction.

#### Edge embedding learning for MPI prediction

Next, we generate the edge embedding of an edge $\left({v}_i^M,{v}_j^P\right)$ connecting a metabolite node ${v}_i^M$ and a protein node ${v}_j^P$, based on the node embedding vectors of the metabolite ${v}_i^M$ and the protein ${v}_j^P$. Five operators are adopted to construct edge embeddings, following [38]: 


(12) \begin{align*}Concatenate: & {h}_{\left({v}_i^M,{v}_j^P\right)}=\left[{h}_i^M\left\Vert{h}_j^P\right.\right]\nonumber\\Hadamard: & {h}_{\left({v}_i^M,{v}_j^P\right)}={h}_i^M\odot{h}_j^P\nonumber\\Average: & {h}_{\left({v}_i^M,{v}_j^P\right)}=\left({h}_i^M+{h}_j^P\right)\Big/2\nonumber\\L1: & {h}_{\left({v}_i^M,{v}_j^P\right)}=\left|{h}_i^M-{h}_j^P\right|\nonumber\\L2: & {h}_{\left({v}_i^M,{v}_j^P\right)}={\left|{h}_i^M-{h}_j^P\right|}^2 \end{align*}

where ${h}_{\left({v}_i^M,{v}_j^P\right)}$ denotes the edge embedding vector of $\left({v}_i^M,{v}_j^P\right)$, and $\odot$ denotes the element-wise Hadamard product operator.

Then, we adopt a multi-layer perceptron (MLP) as the edge classifier to predict links between metabolites and proteins. By taking the edge embedding $ {h}_{\left({v}_i^M,{v}_j^P\right)}, $ as the input, the edge classifier predicts the probability $ {p}_{ij}^{MP} $ of an edge connecting the metabolite $ {v}_i^M $ and the protein $ {v}_j^P $ as: 


(13) \begin{align*} {p}_{ij}^{MP}=\mathrm{Sigmoid}\left(\mathrm{MLP}\left({h}_{\left({v}_i^M,{v}_j^P\right)}\right)\right) \end{align*}

It is worth noting that the MPI prediction can be seen as a binary edge classification task. To construct the whole dataset for MPI prediction, the positive samples are all the edges in the protein–metabolite network, i.e. $\left\{\left({v}_i^M,{v}_j^P\right)\in{\mathcal{E}}^{MP},{v}_i^M\in{\mathcal{V}}^M,{v}_j^P\in{\mathcal{V}}^P\right\}$. While the negative samples are disconnected node pairs between a metabolite and a protein, randomly sampled from the given metabolite node set ${\mathcal{V}}^M$ and the given protein node set ${\mathcal{V}}^P$, i.e. $\left\{\left({v}_i^M,{v}_j^P\right)\notin{\mathcal{E}}^{MP},{v}_i^M\in{\mathcal{V}}^M,{v}_j^P\in{\mathcal{V}}^P\right\}$. In addition, in our experiments, the number of negative samples is set to 10 times of that of positive samples, since in the real-world scenario, the number of unobserved interactions between metabolites and proteins is much larger than that of observed interactions between metabolites and proteins. Then, given the constructed whole dataset, we randomly sample a fraction of positive and negative samples as the training set. Based on the ground-truth binary edge labels and the predicted edge probabilities of all positive and negative samples in the training set, one can easily compute the Binary Cross Entropy loss ${\mathcal{L}}_{bce}$ for binary classification of MPI. Given that negative samples (non-interacting pairs) vastly outnumber positive samples (interacting pairs), to address this imbalanced data issue, we modify the original Binary Cross Entropy loss ${\mathcal{L}}_{bce}$ to a weighted Binary Cross Entropy loss ${\mathcal{L}}_{wbce}$ by assigning larger weight to the positive samples, while smaller weight to the negative samples. The ratio of weight of positive samples over that of negative samples is set to 10 in our experiments.

By combining the weighted Binary Cross Entropy loss ${\mathcal{L}}_{wbce}$ and the heterogeneous neighbor contrastive loss ${\mathcal{L}}_c$, the total loss $\mathcal{L}$ of the proposed HNCGAT is defined as follows:


(14) \begin{equation*} \mathcal{L}={\mathcal{L}}_{wbce}+\lambda{\mathcal{L}}_c \end{equation*}

where $\lambda$ is a weight hyperparameter to balance the effect of ${\mathcal{L}}_c$. The learnable parameters of the proposed HNCGAT are optimized in an end-to-end fashion, using the total loss $\mathcal{L}$ in Equation (14).

## Results and discussion

### Baselines

The proposed HNCGAT was compared with three baselines:

The graph convolutional network (GCN) [39] is a semi-supervised graph neural network that simply averages the neighbors’ embeddings followed by linear projection.

The Heterogeneous Graph Transformer (HGT) [40] employs meta-relation based mutual attention to conduct message passing on heterogeneous graphs and automatically learns the importance of implicit meta paths.

The heterogeneous graph attention networks (HAN) [41] are equipped with hierarchical attentions to effectively gather neighbor information from diverse meta paths.

### Implementation details

The proposed HNCGAT was implemented by PyTorch 1.10.1 [42]. We trained HNCGAT with 1000 epochs by the Adam optimizer [43] with the learning rate of 5e-3. An $\ell 2$-norm regularization was applied on the trainable parameters to prevent overfitting with the weight decay of 1e-5. The number of node embedding dimensions was set to $\mathbb{d}=64$, and the temperature parameter $\mathrm{\tau}$ in the heterogeneous neighbor contrastive loss was set to 0.1. Following the literature on link prediction [21], we adopted two common metrics, i.e. the area under the ROC curve (AUC) and average precision (AP) to evaluate the performance of MPI prediction.

### Performance of HNCGAT

We investigate the performance of HNCGAT for MPI prediction under various training fractions, i.e. 30, 50, and 90%. For each training fraction, we repeated random split five times, and reported the mean and standard deviation of the AUC and AP scores over the five random splits for all comparing methods. Table 3 shows the MPI prediction results on *Arabidopsis* heterogeneous networked dataset. We have the following observations:

**Table 3:** The performance of HNCGAT with varied training fraction

Operator	Metrics (%)	Trp: 0.3				Trp: 0.5				Trp: 0.9			
		HGT	GCN	HAN	HNCGAT	HGT	GCN	HAN	HNCGAT	HGT	GCN	HAN	HNCGAT
Concatenate	AUC	90 (0.4)	91.1 (2)	87.6 (3.4)	**93.8** **(0.6)**	90.1 (0.5)	93.1 (0.7)	81.5 (8.2)	**95.5** **(0.3)**	90.9 (0.3)	93.9 (2)	87.6 (3.3)	**97.3** **(0.4)**
	AP	65.6 (0.6)	63.9 (2.6)	43.4 (1.9)	**71.2** **(4.4)**	65.5 (0.8)	65.4 (3.4)	36.9 (3.4)	**77.7** **(1.6)**	65.5 (0.8)	69.1 (2.6)	44.6 (2.6)	**81.9** **(2.1)**
L1	AUC	90 (0.3)	91.3 (2.7)	87.3 (3)	**93.2** **(0.7)**	90.3 (0.1)	93.6 (0.7)	88.9 (1.2)	**93.8** **(1.4)**	91.2 (0.2)	91.9 (3.9)	87.2 (5.6)	**96** **(0.8)**
	AP	65.9 (0.5)	64.8 (3.2)	46 (2.1)	**69.6** **(3.7)**	66.1 (0.6)	68.2 (1.1)	47.5 (4.3)	**69.9** **(6.1)**	66.6 (0.7)	65.3 (7.1)	45.9 (3.4)	**76.8** **(2.4)**
L2	AUC	90 (0.4)	79.5 (15.1)	89.3 (1.4)	**93.8** **(0.6)**	90.5 (0.2)	80.9 (15.5)	90.6 (1.9)	**95.1** **(0.2)**	91.1 (0.4)	80.9 (15.6)	92.7 (1.8)	**96.7** **(0.6)**
	AP	66.3 (0.5)	59.8 (3.8)	49 (4.3)	**70.9** **(1.9)**	66.7 (0.5)	56 (11)	46.9 (3.2)	**73.3** **(3.2)**	67.1 (0.7)	62.3 (4.4)	49.8 (2.2)	**79.5** **(1.4)**
Hadamard	AUC	89.8 (0.2)	86.7 (1.1)	87 (4.8)	**91.1** **(0.9)**	90.3 (0.3)	90.5 (2.1)	88.6 (4.8)	**93.6** **(0.6)**	90.6 (0.3)	95 (0.2)	89.2 (6)	**96.1** **(0.2)**
	AP	**66.1** **(0.5)**	59.9 (2)	49.5 (8.3)	**65.6** **(2.9)**	66.5 (0.7)	66.5 (2.7)	49.8 (9.1)	**73** **(1.5)**	66.1 (0.3)	72.4 (0.7)	48.5 (12.2)	**79.2** **(1.1)**
Average	AUC	89.5 (0.2)	90.8 (2.3)	86.8 (0.9)	**93.1** **(0.4)**	90.1 (0.3)	91.8 (2.6)	84.1 (3)	**94.5** **(0.5)**	90.4 (0.3)	92.4 (4.7)	88.2 (2.9)	**96** **(0.7)**
	AP	64.9 (0.6)	61.8 (3.8)	42.5 (2.9)	**70** **(1.7)**	65.6 (0.7)	66.3 (3.5)	42.9 (2.7)	**73.4** **(2.1)**	65 (0.4)	65.6 (8.5)	46.7 (4.9)	**75** **(3.7)**

The numbers in parentheses are the standard deviations over five random initializations. The best results are in bold.

First, the proposed HNCGAT consistently outperforms all baselines with various edge operators by a large margin in terms of AUC and AP under large training fraction, i.e. 50 and 90%. For example, when the training fraction is 90% and the edge operator is Concatenate, our HNCGAT improves over the second-best method, i.e. GCN by an absolute 2.2 and 12% in terms of AUC and AP. This might benefit from our proposed novel heterogeneous neighbor contrastive learning mechanism, which treats the heterogeneous neighbors connected by various edge types as the positive samples of the anchor node, and the heterogeneous non-neighbors as the negative samples of the anchor node. By optimizing the heterogeneous neighbor contrastive loss, the positive samples would be pulled close to the anchor node and the negative samples would be pushed far apart from the anchor node. As a result, the node embeddings of proteins, metabolites, and functional-annotations guided by such heterogeneous neighbor contrastive learning can preserve the heterogeneous network topological structure of various edge types, which is beneficial for MPI prediction on *Arabidopsis* heterogeneous network.

Second, under small training ratio, i.e. 30%, our HNCGAT significantly outperforms all baselines in terms of AUC and AP with four edge operators, i.e. Concatenate, L1, L2, and Average. When the edge operator is Hadamard, our HNCGAT achieves the highest AUC but lower AP than HGT. This might be because under small training ratio, our HNCGAT is more suitable with the Concatenate, L1, L2, and average edge operators.

Third, we can observe that GCN with the L2 edge operator would significantly underperform the other four edge operators under various training ratios. While compared with GCN, our HNCGAT can achieve more stable performance with various edge operators under various training ratios. This reflects that GCN is less robust against various edge operators for MPI prediction on *Arabidopsis* heterogeneous network. This might be because GCN simply averages the neighbors’ embeddings followed by linear projection, which treats each neighbor equally during neighborhood aggregation. In contrast, our HNCGAT adopts the graph attention mechanism to learn various importance degree of different neighbors, which yields more robust node embeddings.

### Ablation study

In this section, we perform ablation studies to validate the efficacy of each key component in our proposed HNCGAT model. As shown in Table 4, firstly, excluding the heterogeneous neighbor contrastive loss yields a considerable decline in the performance of HNCGAT with various edge operators. This reflects the importance of the heterogeneous neighbor contrastive loss on guiding the embeddings of proteins, metabolites, and functional-annotations to effectively preserve the heterogeneous network topological structures in the *Arabidopsis* heterogeneous network. Secondly, when learning the embedding of proteins, without utilizing the information of protein attributes, protein–protein-interactions and protein–functional-annotation-associations, all lead to worse performance than HNCGAT. Similarly, for node embedding learning of metabolites, removing the information of metabolites attributes, metabolite–metabolite-structure-similarities, and metabolite–functional-annotation-associations, all degrade the performance of HNCGAT. This demonstrates that taking full advantage of the multimodal information of heterogeneous topological structures and semantics in the *Arabidopsis* heterogeneous network is indeed essential for learning informative node embeddings of proteins and metabolites. Furthermore, among all the multimodal information, without utilizing the metabolite structure similarity emerges as the most influential factor. This can be attributed to the fact that the metabolite structure similarity constitutes the majority of links in the *Arabidopsis* heterogeneous network, as shown in Figure 1. Removing this component would consequently lead to substantial information loss in predicting the MPIs.

**Table 4:** AUC and AP of HNCGAT variants

Operator	Metrics	HNCGAT	w/o neighbor contrastive loss	w/o protein node attribute	w/o protein– protein interaction	w/o protein functional-annotation association	w/o metabolite node attribute	w/o metabolite structure similarity	w/o metabolite–functional-annotation association
Concatenate	AUC	**97.3** **(0.4)**	95.2 (0.2)	95.8 (0.4)	94.6 (0.3)	95.5 (0.6)	95.3 (0.4)	92.3 (1.3)	95.3 (0.6)
	AP	**81.9** **(2.1)**	69.2 (1.5)	73.3 (2.4)	70 (1.7)	72.5 (2.1)	70.8 (1.5)	51.1 (5.4)	71.3 (2.6)
L1	AUC	**96** **(0.8)**	94.4 (0.4)	94.1 (0.4)	93.7 (0.6)	94.1 (0.4)	94 (0.7)	91.2 (0.9)	94.2 (0.4)
	AP	**76.8** **(2.4)**	62.8 (2.2)	69.1 (2)	65.6 (2.9)	64.3 (1.7)	64.2 (3.4)	50.1 (6.1)	65.6 (2.6)
L2	AUC	**96.7** **(0.6)**	93.9 (0.4)	90.1 (0.7)	92 (2)	89.6 (0.4)	89.1 (0.7)	90.8 (1.7)	87.5 (2.7)
	AP	**79.5** **(1.4)**	65.8 (3.4)	52.9 (3.1)	55.1 (6.9)	50.8 (2.3)	49.3 (2.4)	50.2 (2.8)	49.7 (1.5)
Hadamard	AUC	**96.1** **(0.2)**	93.3 (2.4)	94.5 (0.6)	94.2 (0.5)	94.7 (0.4)	94.6 (0.6)	93.3 (0.8)	94.6 (0.9)
	AP	**79.2** **(1.1)**	62.6 (7.5)	68.2 (2.3)	68.2 (3)	68.5 (2.2)	68.4 (2.4)	58.4 (5.2)	70.3 (4.5)
Average	AUC	**96** **(0.7)**	93.3 (0.5)	93.1 (0.4)	93.4 (0.5)	93.4 (0.5)	92.6 (0.5)	91.2 (1.3)	92.4 (0.4)
	AP	**75** **(3.7)**	68 (3)	70.1 (2.4)	68.3 (1.8)	68 (2.7)	68.3 (2.3)	52.8 (3.6)	68.1 (2.6)

The numbers in parentheses are the standard deviations over five random initializations. The best results are in bold.

### Hyperparameter analysis

We study the sensitivity of HNCGAT to the hyperparameters 𝕕 , $\lambda $, $\tau $.

**Figure 4:**
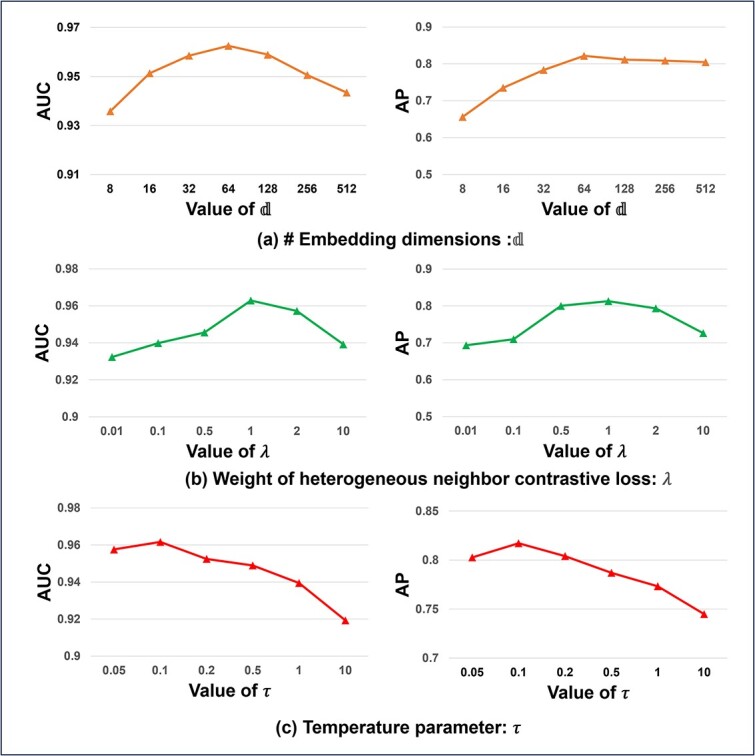
Parameter sensitivity of HNCGAT: (a) The number of embedding dimensions (b) The weight of heterogeneous neighbor contrastive loss (c) The temperature parameter.

The hyperparameter 𝕕 is the number of embedding dimensions. As shown in Figure 4a, both higher AUC and AP can be obtained, as 𝕕 is increased from 8 to 64. While when 𝕕 is further increased, both AUC and AP exhibit a decreasing trend. Thus, it is suggested to set $\mathbb{d}=64$ in our HNCGAT model.

The hyperparameter $\lambda$ represents the weight of heterogeneous neighbor contrastive loss ${\mathcal{L}}_c$. As illustrated in Figure 4b, the optimal performance in terms of both AUC and AP is achieved when $\lambda$ approaches 1. This observation implies that maintaining a balance where the ratio of the heterogeneous neighbor contrastive loss ${\mathcal{L}}_c$ to the Binary Cross Entropy loss ${\mathcal{L}}_{bce}$ is ~ 1:1 leads to enhanced performance in our HNCGAT model.

The hyperparameter $\tau$ is the value of the temperature parameter of heterogeneous neighbor contrastive loss ${\mathcal{L}}_c$. As illustrated in Figure 4c, the optimal performance in terms of both AUC and AP is obtained when $\tau$ approaches 0.1, while larger $\tau$ leads to lower results. Thus, it is suggested to set $\tau$=0.1 in our HNCGAT model.

### Visualization

**Figure 5:**
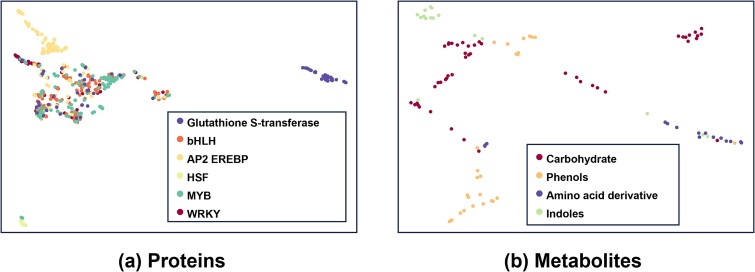
Visualization of (a) protein and (b) metabolite embeddings from six protein families and four metabolite categories.

In this section, to illustrate the biological relevance of the learned embeddings, we visualized the embeddings of 512 proteins from six families and 105 metabolites from four categories using the UMAP method [44] in Figure 5. The protein visualization result in Figure 5a shows that the Glutathione S-transferase family can differ from other transcript factor families (MYB, WRKY, AP2 EREBP, HSF, and bHLH). And some proteins in HSF, AP2 EREBP, and MYB family can be clustered from other protein families. More clearly, in metabolite visualization result Figure 5b, 105 metabolites can form clusters between different known classes. These results reflect the underlying biological relevance of learned embeddings of proteins and metabolites.

### Case study

**Figure 6:**
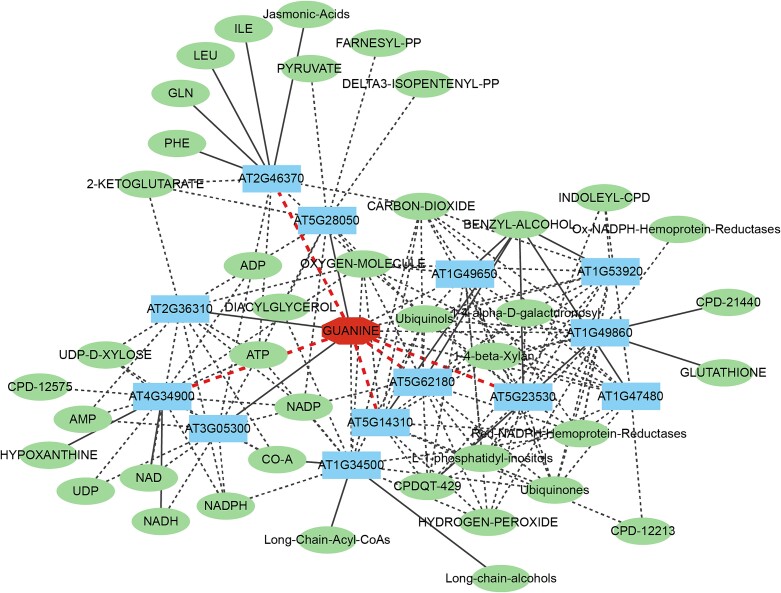
The protein–metabolite network of guanine.

In this section, we initiate a case study focusing on proteins that interact with the metabolite guanine, to evaluate the predictive capacity of HNCGAT. By leveraging the optimal hyperparameter configurations outlined in the implementation details, we utilize the known MPIs as the training set while employing the unknown MPIs as the candidate set. Subsequently, once HNCGAT provides the predicted probability of interaction for metabolite guanine with all protein candidates, we proceed to rank these proteins based on the aforementioned predicted probability. Consequently, the top 10 proteins, shown in Table 5, represent the most probable candidates interacting with the metabolite guanine. We further construct the protein–metabolite network of guanine, which consists of the protein–metabolite interactions predicted by our method or included in the database, in Figure 6. As shown in the figure, the network also contains the second-degree neighbors of guanine, which are the metabolites interact with the proteins. In Figure 6, the metabolite guanine is at the center and is represented by an octagon. The proteins connected to guanine are depicted as rectangles, with solid lines indicating the metabolite–protein associations that are included in the database, and dashed lines representing the associations predicted by our model. Among these, the bold dashed lines represent the MPIs that we have found through literature in our case study. We have also listed the associations of these proteins with other metabolites (represented by ellipses).

**Table 5:** Top 10 proteins most probably interact with the metabolite guanine predicted by our HNCGAT model

Gene locus	Gene name	Reference	Predicted score
AT1G49860	AtGSTF14, glutathione s-transferase (class phi) 14	N.A.	0.64
AT2G46370	JAR1, Jasmonate Resistant 1	[40, 41]	0.56
AT1G47480	Alpha/beta-hydrolases superfamily protein	N.A.	0.56
AT4G34900	AtXDH2, Xanthine Dehydrogenase 2	[42, 43, 44]	0.56
AT1G34500	MBOAT (membrane bound O-acyl transferase) family protein	N.A.	0.55
AT5G62180	Carboxyesterase that binds strigolactones	[39]	0.55
AT5G14310	Carboxyesterase 16	[39]	0.54
AT1G49650	Alpha/beta-Hydrolases superfamily protein	N.A.	0.54
AT1G53920	Contains lipase signature motif and GDSL domain	N.A.	0.53
AT5G23530	Carboxyesterase 18	[39]	0.53

Guanine is one of the primary components of DNA. Previous research on guanine has been conducted mainly in the medical field: mycophenolate mofetil (an immunosuppressant used to prevent organ rejection after transplantation) is metabolized into mycophenolic acid by carboxylesterases; the latter inhibits the synthesis of guanine nucleotides through the *de novo* purine synthesis pathway [45]. This implies guanine may interact with the carboxylesterases such as AT5G14310, AT5G62180, and AT5G23530 predicted by our HNCGAT model.

Guanine is also the most easily oxidized base [46]. Recently, scientists found that guanine enhanced plant resistance to pathogens required AtJAR1 (AT2G46370 predicted by our HNCGAT model) in *Arabidopsis* [47]. Guanosine is deaminated to xanthosine by guanosine deaminase. In plants, xanthosine can only be degraded and not be salvaged into nucleotides [48]. AT4G34900 is xanthine dehydrogenase 2 and involve in xanthine metabolism process [49, 50], which implies the interaction with guanine predicted by our HNCGAT model.

### Comparison with co-expression method

Schläpfer *et al*. [16] presented a co-expression method to identify metabolic enzymes, pathways, and gene clusters from a sequenced genome, which is typically used for predicting MPIs. However, they utilized the transcriptome and genome data to predict the MPIs, in contrast, our HNCGAT utilized heterogeneous graph data to predict the MPIs. Due to different types of input data, it is infeasible to evaluate the co-expression model in Table 3, as our HNCGAT and other graph neural network baselines.

Instead, we compared the MPIs predicted by Schläpfer *et al*. with our HNCGAT, the detailed comparative results are shown in Supplement Table 1. There are 9026 MPIs predicted by Schläpfer *et al*., among them, 7498 (nearly 83%) MPIs have already been included in the input data of our HNCGAT, which was collected in AraCyc database. For the remaining 1528 MPIs predicted by Schläpfer *et al*. (but not included in the input data of our HNCGAT), 1405 MPIs (nearly 92%) can be effectively predicted by our HNCGAT.

Since Schläpfer *et al*.'s work, which served as a data source for AraCyc, was published in 2017, and the AraCyc database has been updated many times since then, our HNCGAT's input data cannot fully cover the MPIs predicted by Schläpfer *et al*. However, the vast majority of remaining MPIs predicted by Schläpfer *et al*. (but not included in the input data of our HNCGAT) can be effectively predicted by our model, demonstrating the robustness of our HNCGAT model for MPI prediction.

## Conclusion

The prediction of MPIs plays an important role in plant basic life functions. Compared with the traditional experimental methods and the high-throughput genomics methods using statistical correlation, applying heterogeneous graph neural networks to the prediction of MPIs in plants can reduce the cost of manpower, resources, and time. However, to the best of our knowledge, applying heterogeneous graph neural networks to the prediction of MPIs in plants still remains under-explored. In this study, we construct an *Arabidopsis* heterogeneous network using multiple public databases, which contains three types of nodes (i.e. proteins, metabolites, and functional-annotations) and five types of edges (i.e. metabolite–protein-interactions, metabolite–functional-annotation-associations, metabolite–metabolite-structural similarity, protein–protein-interactions, and protein–functional-annotation-associations). To predict MPIs in *Arabidopsis*, we propose a novel HNCGAT model. The proposed HNCGAT firstly employs a heterogeneous graph attention network to learn node embeddings of proteins, metabolites, and functional-annotations by the type-specific attention-based neighborhood aggregation mechanism. Then, HNCGAT devises a heterogeneous neighbor contrastive learning framework to guide the node embeddings to preserve the heterogeneous network topological structures of diverse edge types in the *Arabidopsis* heterogeneous network. Extensive experimental results and ablation study demonstrate the effectiveness of the proposed HNCGAT model for MPI prediction in *Arabidopsis*. In addition, a case study on our MPI prediction results supports that the proposed HNCGAT model can effectively predict the potential MPIs, which have not yet included in the public plant metabolite database AraCyc, but have been identified by the recent literature. In conclusion, the present study provides a powerful tool to infer MPIs in plant.

In our proposed heterogeneous neighbor contrastive learning framework, minimizing the neighbor contrastive loss would map the anchor node close to its heterogeneous neighbors and far away from its heterogeneous non-neighbors. One limitation of the framework is that all the heterogeneous neighbors of the anchor are treated equally in the current neighbor contrastive loss. To improve the current framework, a future direction is to propose a weighted neighbor contrastive loss to assign adaptive weights to different heterogeneous neighbors to reflect their various importance degree toward the anchor node.

**Key Points**

- The prediction of metabolite-protein-interactions (MPIs) plays an important role in plant basic life functions. Compared with the traditional experimental methods and the high-throughput genomics methods using statistical correlation, applying heterogeneous graph neural networks to the prediction of MPIs in plants can reduce the cost of manpower, resources, and time. However, to the best of our knowledge, applying heterogeneous graph neural networks to the prediction of MPIs in plants still remains under-explored. In this work, we propose a novel model named HNCGAT, for the prediction of MPIs in *Arabidopsis*.
- We construct the first *Arabidopsis* heterogeneous networked dataset, which integrates multimodal information from three types of nodes and five types of edges.
- We propose a novel HNCGAT model for MPI prediction in *Arabidopsis*. The proposed HNCGAT employs the type-specific attention-based neighborhood aggregation mechanism to learn node embeddings. Moreover, HNCGAT designs a novel neighbor contrastive learning framework to guide node embedding learning to preserve the heterogeneous network topologies for effective MPI prediction.
- Extensive experimental results and ablation study demonstrate the effectiveness of the proposed HNCGAT for MPI prediction in *Arabidopsis*. In addition, a case study on our MPI prediction results supports that the proposed HNCGAT model can effectively predict the potential MPIs that have not yet included in the public plant metabolite database AraCyc, but have been identified by the recent methods in the literature.

## Supplementary Material

Supplement_table_1_bbae397

## Data Availability

Source code and data can be downloaded from https://github.com/xzhouhainanu/HNCGAT/.
